# Amino‐ and Thiol‐Functionalized Brewer's Spent Yeast Hydrolysates: Physicochemical and Antioxidant Properties

**DOI:** 10.1002/fsn3.71164

**Published:** 2025-11-11

**Authors:** Elahe Abedi, Philip C. Wietstock, Brian Gibson

**Affiliations:** ^1^ Department of Food Science and Technology, Faculty of Agriculture Fasa University Fasa Iran; ^2^ Technische Universität Berlin, Institute of Food Technology and Food Chemistry Chair of Brewing and Beverage Technology Berlin Germany

**Keywords:** amination, food protein modification, functionalization, pepsin hydrolysis, thiolation

## Abstract

A significant by‐product of beer brewing is brewer's spent yeast (BSY), which is often discarded. However, it has been demonstrated that the feasibility of BSY valorization can be achieved through the extraction and isolation of a range of economically valuable components, including proteins, functional peptides and amino acids, vitamins, bioactive β‐glucans, minerals, flavor compounds, and dietary fiber. In the present study, BSY valorization was conducted through hydrolysis with pepsin to obtain HBSY with varying degrees of hydrolysis (DH%). These hydrolysates were then thiolated and aminated using different reagents, including silanization, amidation, thiourea, and glutaraldehyde cross‐linking. According to Ellman's reagents, the thiolation (SH; 104.16 mg/100 g for HBSY and 39.37 mg/100 g for BSY) and ninhydrin reagent, amination (NH) contents of BSY (16.66 mg/100 g) and HBSY (41.16 mg/100 g) in silanization were substantially higher than those of other treatments. FTIR depicted an absorption peak around 2048 cm^−1^ (~45%) and 2347–2438 cm^−1^ after thiolation, and an augmented N—H bond at 1033 cm^−1^ and 1625 cm^−1^ following amination. The amid bands I and II underwent significant changes following thiolation and amidation, which were confirmed by SEM images. CD spectroscopy revealed that the amount of α‐helix reduced and converted to random coil structure. A positive correlation (*R* = 0.99, *p* < 0.05) was observed with %DH, thiol, and amino surface groups, whereas a negative correlation was observed between surface hydrophobicity (*H*_0_) and thiol (*R* = −0.94, *p* < 0.05) and amino groups (*R* = −0.97, *p* < 0.05). The thiolated and aminated HBSY significantly enhanced emulsion (78.1% and 91.6%) and foam‐forming abilities (83.5% and 98%), respectively, which were more pronounced at basic and acidic pH, respectively. Moreover, the antioxidant capacities (DPPH radical scavenging activity) substantially improved after thiolation (33.6%–79.1%) and amination (21.9%–51.7%) compared to BSY (5.6%–11.7%) and HBSY (12.9%–24.6%). Therefore, the thiol‐ and amino‐functionalization of HBSY allows for the development of novel applications, specifically as a functional food ingredient and as a substrate for fermentation processes.

## Graphical abstract

A significant by‐product of beer brewing is brewer's spent yeast (BSY), which is often discarded. In the present study, BSY valorization was conducted through hydrolysis with pepsin to obtain HBSY with varying degrees of hydrolysis. These hydrolysates were then thiolated and aminated using different reagents, including silanization, amidation, thiourea, and glutaraldehyde cross‐linking. Results showed that the thiol‐ and amino‐functionalization of HBSY allows for the development of novel applications, specifically as a functional food ingredient and as a substrate for fermentation processes.

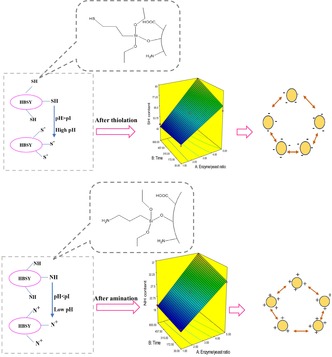

**Abbreviations**
ANP: amination of hydrolyzed yeast by amidation
ANS: 8‐anilino‐1‐naphthalenesulfonic acid ammonium salt
ANY: amination of yeast by amidation
APTES: 3‐aminopropyltriethoxysilane
ASY: thiolation of yeast by amidation
BSY: brewers' spent yeast
BSY: Brewer's spent yeast
DH: degrees of hydrolysis
DMF: *N*,*N*‐dimethylformamide
EDC: 1‐ethyl‐3‐(3‐dimethyl aminopropyl) carbodiimide hydrochloride
GNP: amination of hydrolyzed yeast by glutaraldehyde cross‐linking
GNY: amination of yeast by glutaraldehyde cross‐linking
GRAS: generally recognized as safe
GSP: thiolation of hydrolyzed yeast by amidation
GSP: thiolation of hydrolyzed yeast by glutaraldehyde cross‐linking
GSY: thiolation of yeast by glutaraldehyde cross‐linking
HBSY: hydrolyzed BSY
MPTES: mercaptopropyltriethoxysilane
NH: amination
NHS: *N*‐hydroxysuccinimide
NHS: *N*‐hydroxysuccinimide
PEI: polyethyleneimine
SH: thiolation
SNP: amination of hydrolyzed yeast by silanization
SNY: amination of yeast by silanization
SSP: thiolation of hydrolyzed yeast by silanization
SSY: thiolation of yeast by silanization
TSP: thiolation of hydrolyzed yeast by thiourea
TSY: thiolation of yeast by thiourea

## 1 Introduction

Yeast plays a crucial role in producing beer by converting sugars from grains into alcohol. Brewer's spent yeast (BSY), or residual yeast, is a wort by‐product of the brewing process. BSY has been recognized as Generally Recognized As Safe (GRAS) for human consumption, containing abundant B vitamins, minerals, proteins, and high‐content nucleic acids (Puligundla et al. 2020). Due to the degradation of yeast cells, numerous compounds are also produced, which serve as valuable additives in food products that are rich in free amino acids, β‐glucans, mannoproteins, peptides, and antioxidant properties, contributing additional properties to food formulations (Heppner and Livney 2025). These play a significant role in textural stabilization, as well as serving as flavoring compounds, emulsifiers, and functional ingredients, alongside potential health benefits via bioactive peptides (Li et al. 2022). Due to its abundance of nutrients, wide availability, and low cost, BSY is commonly used in animal feed and aquaculture formulations (Jaeger et al. 2020; Puligundla et al. 2020).

The substantial environmental and economic consequences of global food waste have spurred the development of numerous initiatives aimed at managing waste throughout the supply chain (Heppner and Livney 2025; Soon et al. 2025). BSY makes up 1.5%–2.5% of the overall beer production volume, which becomes ineffective and requires disposal (Jaeger et al. 2020; Podpora et al. 2016). In some cases, due to specific disadvantages associated with BSY, including limited shelf life, high transportation costs, and the need for further processing, this material is often discarded into the environment. For these reasons, a large number of studies employing various valorization strategies for BSY through the extraction and isolation of various economically valuable components, including proteins, functional peptides and amino acids, vitamins, bioactive β‐glucans, minerals, flavor compounds, and fiber (Jaeger et al. 2020; Oliveira et al. 2022; Puligundla et al. 2020).

Thiomer‐based polymers represent a promising multifunctional class, which involves disulfide bridge formation—both inter‐ and intramolecular—between polymer‐bound thiol groups and cysteine‐rich components (Abedi, Akhavan, et al. 2023; Abedi, Savadkoohi, and Banasaz 2023; Leichner et al. 2019). Aminated polymers are also valuable for their versatility and the ease of introducing amino functionalities (Abedi, Akhavan, et al. 2023; Fatima et al. 2023). A range of benefits associated with thiolated and aminated polymers has been documented in both drug delivery, where enhanced mucoadhesion is central, and diverse biomedical areas—encompassing therapeutics, diagnostics, and regenerative medicine (Lobo et al. 2021).

The influence of limited hydrolysis using different proteases on protein autolysate has been investigated from the perspectives of its structural, functional, antioxidant, and interfacial rheological properties. For instance, multiple protein substrates, including brewers' spent grain (Celus et al. 2007), hempseed protein (Wang et al. 2025), soybean protein isolate (Cui et al. 2013; Gao et al. 2024; Yuan et al. 2012), walnut protein (Feng et al. 2024), glutelin‐1 (Wang, Wang, et al. 2021), egg yolk (Bao et al. 2017), and lentil protein concentrate (Vogelsang‐O'Dwyer et al. 2023), have been studied by different protease enzyme sources. On the other hand, in the studies conducted by Mfoafo et al. (2023), Afshar and Ghaee (2016), and Omer et al. (2021), the drug delivery properties and mucoadhesive properties of thiolated and aminated polymers have been investigated. However, the thiol‐ and amino‐functionalized BSY, or its hydrolyzed form (HBSY), has not yet been evaluated. The aim of this study is to valorize BSY through thiolation and amidation, and then compare its physicochemical and antioxidant properties with those of BSY and HBSY. To achieve this goal, pepsin was used to produce the enzymatic hydrolysis of BSY with varying degrees of hydrolysis. Thiol‐functionalized BSY/HBSY samples were subjected to modification via silanization, amidation, thiourea conjugation, and glutaraldehyde cross‐linking. Moreover, amino‐functionalized BSY/HBSY was yielded through silanization, amidation, and glutaraldehyde cross‐linking. The influence of varying surface hydrophobicity and hydrolysis degrees on the extent of modification was investigated. Spectrophotometry, FTIR, and CD spectrometry were used to characterize the thiol and amino surface. The functional (water‐holding capacity, emulsifying capacity/stability, and foaming capacity/stability) and antioxidant properties of thiol‐ and amino‐functionalized BSY/HBSY were compared to those of HBSY and BSY without any modification.

## 2 Materials and Methods

### 2.1 Chemical Composition of Brewer's Yeast

Brewers' spent yeast ( *Saccharomyces cerevisiae* yeast) produced at TU Berlin consisted of 8% moisture, 6.9% fat, 41% protein, 2.6% crude fiber, and 12.7% ash. Carbohydrate content (28.4%) was determined by difference. Chemical compounds were measured by the AOAC methods 960.39, 979.09, 962.09, and 923.03.

Whole chemical compounds, including mercaptopropyltriethoxysilane (MPTES), *N*‐hydroxysuccinimide (NHS), 1‐ethyl‐3‐(3‐dimethyl aminopropyl) carbodiimide hydrochloride (EDC), polyethyleneimine (PEI), 8‐anilino‐1‐naphthalenesulfonic acid ammonium salt (ANS), 3‐aminopropyltriethoxysilane (APTES), and *N*,*N*‐dimethylformamide (DMF) were supplied by Thermo Fisher Scientific Co., Limited (Berlin, Germany). All of the reagents used were of analytical grade. Deionized water was used for the experiments.

### 2.2 Limited Enzymatic Hydrolysis of BSY by Pepsin and Determination of the Degree of Hydrolysis

The hydrolyzed yeast was produced using different enzyme/substrate ratios (1%–5%), temperatures (35°C–55°C), and times (30–600 min) (Table 1). Following the reaction (pH adjusted to 3.5 with 2.0 M citric acid), the enzyme was inactivated by adjusting the pH to 7 and 90°C for 5 min. Centrifugation (5000 *g*, 20 min, 4°C) yielded a supernatant and pellet, which were individually freeze‐dried (Dol, USA).

**TABLE 1:** Experimental design for response surface methodology.

Parameters	−1	0	+1
Enzyme/substrate ratios (%)	1	3	5
Temperatures (°C)	35	45	55
Time (min)	30	315	600

The DH of the HBSY was obtained using the pH‐stat method described by Feng et al. (2024). A constant pH during pepsin hydrolysis of the BSY was achieved through a continuous titration with a 0.5 M NaOH solution; the volume of titrant consumed served as a measure of the hydrolysis. The DH (Equation 1) was then calculated.

(1) $$\mathrm{DH}=\frac{B\times N_{b}\times 100}{\alpha \times M_{P}\times h_{\mathrm{tot}}}$$

where *B* is the volume (mL) of NaOH solution consumed, *N*_b_ is the concentration (mol/L) of NaOH standard solution, *α* is the average degree of dissociation of the α‐amino group of the HBSY, *M*_P_ is the mass (g) of substrate protein, and *H*_tot_ is the total number of peptide bonds in the substrate protein (8.0 mmol/g).

### 2.3 Surface Hydrophobicity and Intrinsic Fluorescence Spectra Analysis

Following the method of Jaeger et al. (2023), surface hydrophobicity was assessed. A series of dilutions of the protein dispersions was prepared in 10 mM phosphate buffer (pH 7), a concentration range of 0.0006%–0.015% (w/v). To each 2 mL dilution, 10 μL of ANS (8.0 mM in 0.2 M phosphate buffer, pH 7) was added. The samples were subsequently incubated for 15 min in the dark at 25°C before measurement. Fluorescence emission was measured at 470 nm following excitation at 390 nm (Cytation 5 imaging reader, BioTek Instruments Inc.). Blank measurements were performed without sample dilutions. The results are expressed as the slopes (*R* ^2^ ≥ 0.96) of the linear regression between absorbance and protein concentration (% w/v) after 5 replications.

Fluorescence spectral analysis of the BSY and HBSY was measured through a modified protocol based on Wang, Wang, Chen, et al. (2021). Sample solutions were prepared by dissolving the BSY and HBSY in 10 mM phosphate buffer (pH 7.0) to a final concentration of 0.2 mg/mL. An excitation wavelength of 290 nm and emission wavelengths scanned from 300 to 400 nm with a slit width of 5 nm were used to record fluorescence emission spectra.

### 2.4 Producing Thiol‐Functionalized and Amino‐Functionalized BSY and HBSY

#### 2.4.1 Thiol‐Functionalization by Silanization

**FIGURE 1:**
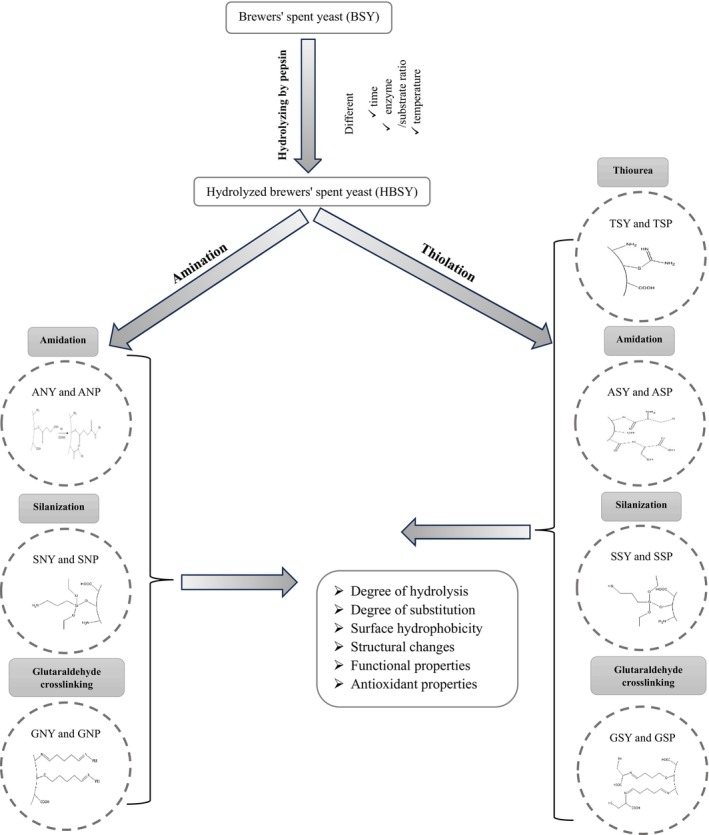
Flow chart of the implemented procedure to produce thiol‐ and amine‐functionalized BSY and HBSY.

After rinsing 1.0 g of BSY and HBSY with 95% ethanol and dispersing them in 30 mL of the same solvent, the pH was adjusted to 4.0 using acetic acid. A solution of 5 mmol MPTES (1.21 mL) was then added dropwise, and the reaction mixture was stirred overnight. The modified BSY and HBSY were then recovered by centrifugation (3600 *g*, 1 min), washed with water, and lyophilized (Dol, USA) (Qiu et al. 2020). The reaction was conducted in duplicate (Figure 1).

#### 2.4.2 Amino‐Functionalization by Silanization

The operation is similar to the thiol modification by silanization described in Section 2.4.1. with the only difference being that 1.21 mL of MPTES is replaced with 1.17 mL (5 mmol) of APTES (Qiu et al. 2020). Reaction was conducted in duplicate (Figure 1).

#### 2.4.3 Thiol‐Functionalization by Amidation

To initiate the reaction, BSY and HBSY (1.0 g) were suspended in 30 mL of deionized water. EDC (0.48 g) and NHS (0.58 g) were added to activate the carboxyl groups, and the pH was adjusted to 5.0. Following 40 min of stirring at room temperature, L‐cysteine (0.606 g, 5 mmol) was added to promote coupling. The pH was adjusted to 7.5 using NaOH (0.5 M), and the reaction was continued for 12 h at room temperature. The resulting product was subsequently washed and lyophilized (Dol, USA) (Qiu et al. 2020). The reaction was conducted in duplicate (Figure 1).

#### 2.4.4 Amino‐Functionalization by Amidation

To achieve amidation modification, the —COOH content on the cell surface must be increased by modifying the hydroxyl groups with an acid anhydride. 1 g of BSY and HBSY were dispersed in 30 mL of DMF, followed by adding 1 mL of pyridine and 2.0 g of succinic anhydride. Following a 12‐h reflux at 80°C, the mixture was washed several times and resuspended in 26 mL of deionized water. EDC (0.48 g) and NHS (0.58 g) were added, and the pH was adjusted to 5.0. After stirring for 40 min at room temperature, a 50% (w/w) aqueous solution of PEI was added to reach a final PEI concentration of 10% (w/w). The pH was then adjusted to 7.5 using NaOH (0.5 M), and the mixture was stirred at room temperature for 12 h. Finally, the mixture was washed and lyophilized (Dol, USA) (Qiu et al. 2020). The reaction was conducted in duplicate (Figure 1).

#### 2.4.5 Thiol‐Functionalization by Glutaraldehyde Cross‐Linking

BSY and HBSY (1.0 g) were suspended in 30 mL of water, to which 0.606 g (5 mmol) of L‐cysteine was incorporated. The mixture was blended for 2 h, after which the yeast cells were collected via centrifugation. The cells were then resuspended in 40 mL of a 2.5% (v/v) aqueous glutaraldehyde solution and agitated for 2 h. Finally, the cells were washed and lyophilized (Qiu et al. 2020). The reaction was conducted in duplicate.

#### 2.4.6 Amino‐Functionalization by Glutaraldehyde Cross‐Linking

BSY and HBSY (1.0 g) were mixed with 30 mL of a 10% (w/w) aqueous solution of PEI and stirred for 2 h. After this, any unreacted PEI was removed through centrifugation. The yeast cells were then resuspended in 40 mL of a 2.5% (w/v) glutaraldehyde aqueous solution and stirred for 2 h. Finally, the cells were washed and lyophilized (Dol, USA) (Qiu et al. 2020). The reaction was conducted in duplicate.

#### 2.4.7 Thiol‐Functionalization by Thiourea

BSY and HBSY (1 g) were combined with thiourea (0.38 g) in a 50 mL flask containing 20 mL of a 1:3 (v/v) hydrochloric acid‐water solution. This mixture was stirred overnight at 25°C. The resulting precipitate was isolated via centrifugation and washed with water to achieve a neutral pH. The washed material was then treated with a NaOH solution (0.5 M) at pH 11.5 and stirred for 6 h. A final wash to neutral pH was followed by lyophilization (Dol, USA) (Qiu et al. 2020). The reaction was conducted in duplicate.

### 2.5 Determination of ‐SH Content

Ellman's method was employed to assess surface thiol content. An aliquot of cell suspension (0.25 mL) was combined with 0.4 M phosphate buffer (0.25 mL, pH 8.0) and Ellman's reagent (0.5 mL, 0.3 g/L). The mixture was incubated at 40°C for 2 h to allow reaction with the surface thiols. After centrifugation to remove the cells, the absorbance of the supernatant at 412 nm was measured using a UV–vis spectrophotometer (PerkinElmer, Lambda 25) against a reagent blank to quantify the released chromophore. The concentration of thiol groups was calculated using a calibration curve established with L‐cysteine, with the equation *y* = 0.003× + 0.0415; *R* ^2^ = 0.9901 (Li et al. 2021).

### 2.6 Determination of ‐NH_2_ Content

Amino group content was assessed using a ninhydrin‐based colorimetric assay. A 1 mL aliquot of cell suspension was combined with 0.5 mL of 0.1 M phosphate buffer (pH 8.0) and 0.5 mL of a 2% ninhydrin solution. The mixture was vortexed, capped, and incubated at 98°C for 15 min. After cooling, centrifugation was used to remove any solids. The resulting supernatant was diluted to 10 mL with deionized water before its absorbance at 570 nm was measured against a reagent blank using a UV–vis spectrophotometer (PerkinElmer, Lambda 25). The concentration of amino groups was measured using a calibration curve established with L‐Lucine with the equation *y* = 0.0326× + 0.0718; *R* ^2^ = 0.9537.

### 2.7 Determination of Isoelectric Point

The calculation of the isoelectric point (pI) was performed by the method of Majzoobi et al. (2012).

### 2.8 Scanning Electron Microscopy (SEM)

SEM images were obtained using a Hitachi S‐2700 at 20 kV.

### 2.9 Fourier Transform Infrared Spectroscopy (FTIR)

FTIR spectra were quantified using a Vertex 70 spectrometer (Bruker Corp., Germany) to obtain the thiol and amino groups on the surface of the BSY and HBSY.

### 2.10 CD Spectroscopy

CD spectra were recorded in the wavelength range of 200 to 260 nm, with a focus on the far‐UV region, using an AVIV spectropolarimeter (model 215, USA). The scans were performed at a rate of 20 nm per minute, maintained at 30°C (Abedi, Torabizadeh, and Orden 2023).

### 2.11 Determination of Antioxidant Activity

#### 2.11.1 DPPH Radical Scavenging Activity

Following the methodology of Xie et al. (2019), Murthy and Naidu (2012) and Hamrouni‐Sellami et al. (2013), the free radical scavenging capacity of samples was evaluated using the DPPH assay. A series of BSY, HBSY, aminated, and thiolated solutions (1.00, 2.00, and 3.00 mg/mL in distilled water) was prepared. Each solution (3 mL) was mixed with DPPH (3 mL, 1 mM). The mixtures were vigorously shaken and incubated at room temperature in the dark for 30 min. The absorbance of each mixture at 517 nm was subsequently measured using a UV–Vis spectrophotometer (PerkinElmer, Lambda 25). The calculation of the scavenging capacity is detailed below:

(2) $$\text{DPPH radical scavenging activity}\;\left(\%\right)=1-\frac{A_{1}}{A_{0}}\times 100$$

*A*_1_: The absorbance of the sample.

*A*_0_: The absorbance of water.

#### 2.11.2 Reducing Power

The reducing power of the BSY, HBSY, and thiolated and aminated HBSY was measured by a modified protocol described by Wang, Wang, Wang, et al. (2021). Samples were prepared at different concentrations (1.00–3.00 mg/mL). An aliquot of each sample (1.0 mL) was combined with phosphate buffer (2.5 mL, 0.2 M and pH 6.6) and potassium ferricyanide (2.5 mL, 1% w/v). The mixtures were incubated (50°C for 20 min). Following incubation, trichloroacetic acid solution (0.5 mL, 10% w/v) was added to precipitate proteins. After centrifugation at 3000 *g* for 10 min, the supernatant (2.5 mL) was incorporated with distilled water (2.5 mL) and an aqueous solution of FeCl_3_ (0.5 mL, 0.1% w/v). After a further 10‐min incubation at 25°C, the absorbance at 700 nm was identified using a UV–Vis spectrophotometer (PerkinElmer, Lambda 25).

#### 2.11.3 Superoxide Anion Radical Scavenging Activity

The hydroxyl radical scavenging capacity of HBSY, thiolated‐ and aminated HBSY was identified using a modified version reported by Zheng et al. (2015). Solutions were prepared at concentrations of 1.00–3.00 mg/mL in distilled water (Zheng et al. 2015). A 2 mL aliquot of each sample was combined with FeSO_4_ (2 mL, 6 mM) and H_2_O_2_ (2 mL, 6 mM). This mixture was incubated at 25°C for 10 min, then salicylic acid (2 mL, 6 mM) was added. After a further 30‐min incubation at 37°C, the absorbance at 510 nm was quantified using a UV–Vis spectrophotometer (PerkinElmer, Lambda 25). The hydroxyl radical scavenging rate was calculated as:

(3) $$\begin{array}{c} \text{Scavenging activity}\;\left(\%\right)=\\ \left(1-\text{absorbance of sample}/\text{absorbance of control}\right)\times 100 \end{array}$$

### 2.12 Measurement of Zeta‐Potential

Zeta‐potential measurements were conducted to assess emulsion stability. The emulsion sample was diluted 1:100 in 2 mL of 10 mM NaCl at pH levels ranging from 4 to 9, and the droplet mobility was measured at 25°C using a Nano‐ZS zetasizer (Malvern, Worcestershire, UK). The reported zeta‐potential is the average of two measurements.

### 2.13 Functional Properties

Functionality measurements of BSY, HBSY, and thiol‐ and amino‐functionalized HBSY were performed by adapting the methodology of Feng et al. (2024) and Comunian et al. (2020). To conduct the solubility test, samples (50 mg) were dispersed in distilled water (20 mL) at different pH levels adjusted to 3, 6, and 9 using 0.5 M HCl/NaOH. The samples were then stirred for 60 min at ambient temperature and centrifuged at 10,000 rpm for 10 min. The Kjeldahl method was used to determine the supernatant protein concentration (AACC, 2000). Solubility was calculated as.

(4) $$\text{Solubility}\;\left(\%\right)=\frac{N_{1}\times 100}{N_{2}}$$

*N*_1_: the protein content of the supernatant,

*N*_2_: the total protein content of the BSY, HBSY, thiolated, and aminated HBSY.

For emulsifying activity, 5 mL of protein solution (0.5%) at pH levels adjusted to 3, 6, and 9 was combined with 5 mL of sunflower oil. The mixture was homogenized at 20,000 rpm for 5 min. Following this, the sample was centrifuged at 1100 *g* for 5 min. The emulsifying activity was then assessed according to Equation (5).

(5) $$\text{Emulsifying activity}\;\left(\%\right)=\frac{H_{1}\times 100}{H_{0}}$$

*H*_1_: the height of the emulsified layer.

*H*_0_: the height of total content.

To assess emulsion stability, the emulsions were heated (80°C for 30 min). Following this heating period, they were centrifuged at 1100 *g* for 5 min. Subsequently, the stability of the emulsion was calculated using Equation (6).

(6) $$\text{Emulsifying activity}\;\left(\%\right)=\frac{H_{2}\times 100}{H_{0}}$$

*H*_2_: the height of the emulsified layer after heating,

*H*_0_: the height of the total‐content emulsified layer before heating.

To assess foaming capacity and stability, the sample suspensions (0.5%) were adjusted to pH levels of 3, 6, and 9, and then vigorously mixed with a laboratory homogenizer at 20,000 rpm for 2 min at ambient temperature, as described in Equation (7).

(7) $$\mathrm{FC}\;\left(\%\right)=\frac{V_{0}}{V}\times 100$$

The volumes of the samples identified foaming stability at 0 and 30 min (Equation 8).

(8) $$\mathrm{FS}\;\left(\%\right)=\frac{V_{30}}{V_{0}}\times 100$$

*V* (mL): the foam volume before homogenization,

*V*_0_ (mL): the foam volume after homogenization and standing for 0 min,

*V*_30_ (mL): the foam volume after homogenization and standing for 30 min.

### 2.14 Experimental Design and Statistical Analysis

The statistical software packages SAS 9.1 (SAS Institute, Cary, NC, USA) and Design Expert (version 6.0.5) were used for the regression analysis of the experimental data and to obtain the regression coefficients (Myers et al. 2016). A Box Behnken design was used to study the effect of three different factors on the DH, NH, SH, and *H*_0_. The factors were enzyme/substrate ratios (1%–5%), temperatures (35°C–55°C), and times (30–600 min) (Table 1). The experimental data were fitted in accordance with Equation (9) as a second‐order polynomial equation, including the linear and interaction effects of each factor:

(9) $$Y=\beta_{0}+\sum_{i=0}^{k}\beta_{i}X_{i}+\sum_{i=1}^{k}\beta_{\mathrm{ii}}{X_{i}}^{2}+\sum_{\begin{array}{c} i=1\\ i<j \end{array}}^{k-1}\sum \beta_{\mathrm{ij}}X_{i}X_{j}$$

where *Y* is the predicted response, *X*_*i*_ and *X*_*j*_ are independent factors, *b*_0_ is the offset term, *b*_*i*_ is the *i*_th_ linear coefficient, *b*_*ii*_ is the *i*_th_ quadratic coefficient, and *b*_*ij*_ is the *ij*_th_ interaction coefficient. All analyses were obtained in triplicate and reported as mean values.

## 3 Results and Discussion

### 3.1 Effects of Enzymatic Hydrolysis on Protein Structure

#### 3.1.1 Degree of Hydrolysis

**FIGURE 2:**
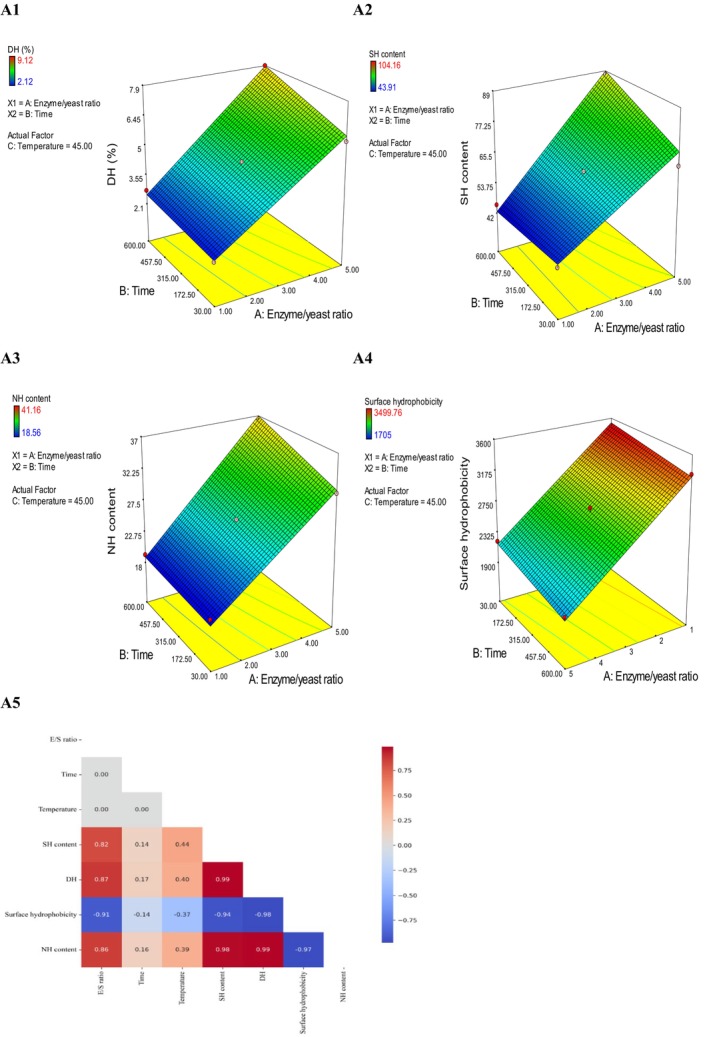
The effect of different E/S ratios, time, and temperature on DH (%) (A1), SH content (A2), NH content (A3), and surface hydrophobicity (A4). The correlation heatmap of E/S ratios, time, and temperature on DH (%), SH content, NH content, and surface hydrophobicity (A5).

Figure 2A presents the DH (%) obtained using pepsin under various enzyme‐to‐substrate ratios (1%–5%), incubation times (10–600 min), and temperatures (35°C–55°C) under optimized pH‐stat conditions (pH 3.5). A 5% (P3), 3% (P2), and 1% (P1) E/S ratio produced DH values of 9.12%, 6.74%, and 3.85%, respectively, after 25 min at 55°C. According to the response surface methodology (RSM), the E/S ratio is the most significant factor influencing DH (%), followed by temperature and time (*A*: E/S ratio, *B*: time, and *C*: temperature).

$$\begin{array}{cc} \mathrm{DH}\;\left(\%\right)= & +4.77+2.34\times A+0.46\times B+1.07\times C+0.31\times A\times B\\ & +0.67\times A\times C+0.082\times B\times C \end{array}$$

In a previous study by Cui et al. (2013), soybean protein hydrolysates were produced using pepsin at various pH levels and hydrolysis times (10–900 min). The authors observed a very slow increase in DH, with the hydrolysis reaction essentially ceasing after 600 min. Consequently, the present study focused on hydrolysis times up to 600 min. Vogelsang‐O'Dwyer et al. (2023) and Segura‐Campos et al. (2012) found that Alcalase, Novozym, and Flavourzyme hydrolyzed lentil and cowpea protein concentrates, respectively, with significantly different enzyme specificities.

#### 3.1.2 The Degree of Surface Substitutions by Thiol and Amino Group

**FIGURE 3:**
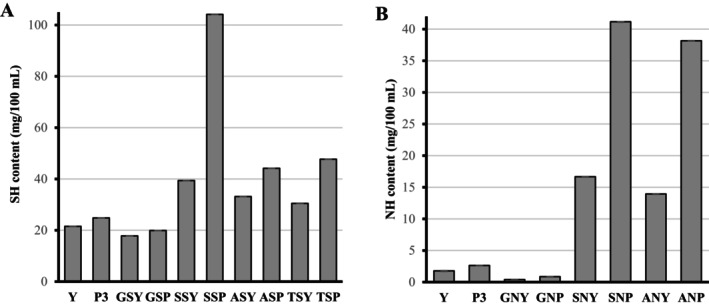
The extent of thiolation (A) and amination (B) by different reagents. ANP (Amination of hydrolyzed yeast by amidation); ANY (Amination of yeast by amidation); ASY (Thiolation of yeast by amidation); GNP (Amination of hydrolyzed yeast by glutaraldehyde cross‐linking); GNY (Amination of yeast by glutaraldehyde cross‐linking); GSP (Thiolation of hydrolyzed yeast by amidation); GSP (Thiolation of hydrolyzed yeast by glutaraldehyde cross‐linking); GSY (Thiolation of yeast by glutaraldehyde cross‐linking); SNP (Amination of hydrolyzed yeast by silanization); SNY (Amination of yeast by silanization); SSP (Thiolation of hydrolyzed yeast by silanization); SSY (Thiolation of yeast by silanization); TSP (Thiolation of hydrolyzed yeast by thiourea); TSY (Thiolation of yeast by thiourea).

The availability and abundance of surface functional groups, including amino, hydroxyl, carboxyl, carbonyl, thiol, and phosphate, make inactivated microorganisms ideal for modification. Modification significantly enhanced the surface thiol and amino content of both BSY and HBSY (Figure 2A). Silanization resulted in the highest increase, reaching 39.37 mg/100 g for BSY and 104.16 mg/100 g for HBSY (Figure 3A). Thiourea yielded 30.5 mg/100 g for BSY and 47.5 mg/100 g for HBSY. Amidation resulted in lower increases to 33.16 and 30.5 mg/100 g values for HBSY and BSY, respectively. Glutaraldehyde cross‐linking, however, reduced thiol group content to 17.83 mg/100 g (BSY) and 19.89 mg/100 g (HBSY). Additionally, silanization significantly enhanced surface amino content in BSY and HBSY (Figure 3B), achieving the highest increases (16.66 mg/100 g for BSY and 41.16 mg/100 g for HBSY). Amidation modification resulted in smaller increases (13.93 mg/100 g for BSY and 38.16 mg/100 g for HBSY), while glutaraldehyde cross‐linking decreased amino content (0.404 mg/100 mL for BSY and 0.86 mg/100 g for HBSY). The extent of the thiol and amino groups on HBSY was significantly greater than that on BSY, which may be associated with a lower *H*_0_ in HBSY compared to BSY. Moreover, more functional groups are available and exposed to react with thiols and amines (see the FTIR Section 2.9).

The higher SH and NH content of the surface after silanization can be attributed to the stability of the —O—Si—S covalent bond, reaction specificity, and pH adaptability. Silanization represents a cost‐effective and efficient technique for the covalent modification of hydroxyl‐rich material surfaces (Boccafoschi et al. 2018). Thiourea forms by a central carbon double‐bonded to sulfur (thione) with two amine groups attached as NH_2_—C(=S)—NH_2_, which is susceptible to decomposition in alkaline environments (Ashiq et al. 2019). Amide bonds are formed through the reaction of a carboxylic acid (or a derivative, such as an acyl chloride) with an amine, often with the aid of heat. It can alter surface hydrophobicity, and amides undergo hydrolysis under strongly acidic or basic conditions, yielding the corresponding carboxylic acid and amine (or ammonia) (Wilding and Micklefield 2012). The authors believe that the probability of degradation of thiol/amine‐functionalized BSY/HBSY by silanization is significantly lower than that of other reagents.

To investigate the relationship between DH (%) and surface thiol/amino content (Figure 2A2), thiolation and amination by silanization were performed at different DH (%). The results showed a strong positive correlation (*r* = +0.99) between the DH (%) and the surface thiol content of HBSY. Similarly, a strong positive correlation (*r* = +0.99) was observed between the DH (%) and surface amino content (Figure 2A3). Based on RSM, an increased E/S ratio, followed by temperature, and time, resulted in a higher surface thiol and amino content (*A*: E/S ratio, *B*: time, and *C*: temperature).

$$\begin{array}{cc} \text{Surface}\;\mathrm{SH}\;\text{content}= & +62.78+18.38\times A+3.08\times B+9.95\times C\\ & +4.69\times A\times B+8.29\times A\times C+0.97\times B\times C \end{array}$$

$$\begin{array}{cc} \text{Surface}\;\mathrm{NH}\;\text{content}= & +26.44+7.97\times A+1.48\times B+3.58\times C\\ & +1.09\times A\times B+2.3\times A\times C+1.48\times B\times C \end{array}$$

#### 3.1.3 Surface Hydrophobicity and Fluorescence Spectroscopy

Figure 2A4 illustrates the relationship between DH (%) and *H*_0_ of BSY and HBSY. Based on the RSM, the E/S ratio, temperature, and time are the primary factors influencing *H*_0_, with the E/S ratio having the most significant effect (*A*: E/S ratio, *B*: time, and *C*: temperature). Moreover, there is a negative relation between *H*_0_ and DH (%). This indicates that a significant decrease in *H*_0_ was observed with increasing DH (%), with the lowest value recorded at DH 9.2%.

$$\begin{array}{cc} H_{0}= & +6355.00-1728.25\times A-258.50\times B-700.25\times C-32.25\times A\times B-137.75\times A\times C\;\\ & -135.25\times B\times C+113.88\times A^{2}+8.88\times B^{2}-166.63\times C^{2} \end{array}$$

This reduction in *H*_0_ may be attributed to the fact that hydrophobic interactions play a central role in protein aggregation. Disruption of hydrophobic interactions within the protein structure initiates aggregation, followed by the reburial of exposed hydrophobic regions through the formation of aggregates. It leads to reduced exposure of hydrophobic amino acid residues to the solvent and the gradual exposure of hydrophilic groups (Abedi et al. 2022; Shuai et al. 2022; Wang et al. 2025). Similar results were observed by Shi et al. (2018) and Feng et al. (2024), who depicted a decrease in the *H*_0_ of walnut protein following limited enzymatic hydrolysis. Pigeon pea protein, lentil, chickpea, after hydrolysis with alcalase or bromelain, exhibited reduced *H*_0_ compared to untreated samples (Xu et al. 2021). Moreover, *H*_0_ of hempseed protein depicted a reducing pattern after hydrolysis with alcalase (Q. Wang et al. 2025). Vogelsang‐O'Dwyer et al. (2023) similarly found that Alcalase and Novozym hydrolysis reduced the *H*_0_ of lentil protein concentrate, supporting the findings presented in this study. However, the exposure of previously buried hydrophobic regions during protein degradation into smaller peptides can increase *H*_0_, as seen in some proteins reported by Konieczny et al. (2020) and Wouters et al. (2016). Jin et al. (2020) have shown that limited hydrolysis of dehulled walnut protein with trypsin increases *H*_0_. This increase may be attributed to the exposure of previously concealed hydrophobic residues resulting from protein unfolding following hydrolysis. The observed variability in *H*_0_ following hydrolysis by different proteases may be associated with differences in enzyme specificity and the resulting variations in the distribution of exposed hydrophobic sites within the peptide fragments.

**FIGURE 4:**
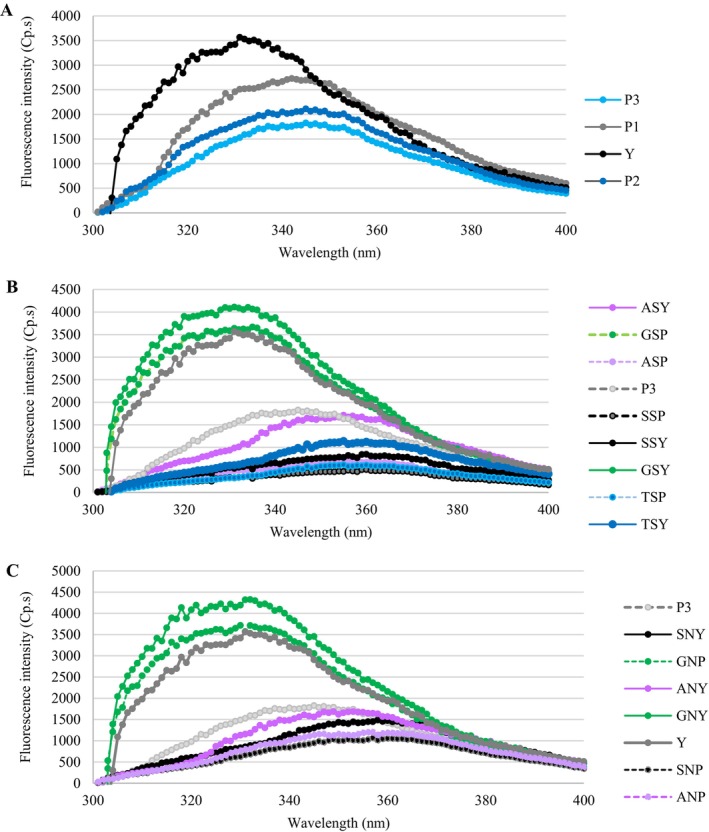
The intrinsic fluorescence spectra of BSY and HBSY (A), thiolation of BSY and HBSY (B), and amination of BSY and HBSY (C). Y, Yeast, P1, Hydrolyzed yeast with 4.6% degree of hydrolysis, P2, Hydrolyzed yeast with 7.8% degree of hydrolysis, P3, Hydrolyzed yeast with 9.2% degree of hydrolysis. ANP, Amination of hydrolyzed yeast by amidation; ANY, Amination of yeast by amidation; ASY, Thiolation of yeast by amidation; GNP, Amination of hydrolyzed yeast by glutaraldehyde cross‐linking; GNY, Amination of yeast by glutaraldehyde cross‐linking; GSP, Thiolation of hydrolyzed yeast by amidation; GSP, Thiolation of hydrolyzed yeast by glutaraldehyde cross‐linking; GSY, Thiolation of yeast by glutaraldehyde cross‐linking; SNP, Amination of hydrolyzed yeast by silanization; SNY, Amination of yeast by silanization; SSP, Thiolation of hydrolyzed yeast by silanization; SSY, Thiolation of yeast by silanization; TSP, Thiolation of hydrolyzed yeast by thiourea; TSY, Thiolation of yeast by thiourea.

Protein conformational changes can be effectively characterized using fluorescence spectroscopy (Guo et al. 2022). Analysis of intrinsic fluorescence spectra provides information on changes in protein tertiary conformation and the polarity of the microenvironment surrounding aromatic amino acids, such as tryptophan (Vera et al. 2019; Wang et al. 2025). An emission peak was observed at approximately 330–334 nm. As DH (%) increased, the fluorescence intensity of HBSY decreased, indicating a reduction in its hydrophobicity. Increasing the DH (%) induced a red shift in λ_max_ to values ranging from 342 to 345 nm (Figure 4). This observation implies that enzymatic hydrolysis leads to protein unfolding, exposing tryptophan residues to a more polar microenvironment (Zhao et al. 2011). This reduction is attributed to several possible reasons: (1) the progressive incorporation of hydrophobic groups into the protein's interior as DH (%) increases (Guo et al. 2022). (2) a reduction in energy transfer from tyrosine to tryptophan residues and/or an increase in nearby fluorescence‐quenching groups (Wang et al. 2025). Various protein substrates support the present observation after hydrolysis through different protease sources, such as the peanut protein (Zhao et al. 2011), walnut protein (Feng et al. 2024), quinoa proteins (Wang, Wang, et al. 2022), and abalone foot muscle proteins (Li et al. 2023). After thiolation and amination, the *H*_0_ decreased, which is in good agreement with the intrinsic fluorescence results. The denaturation‐induced unfolding of proteins by thiolation and amination exposes apolar tryptophan residues, previously sequestered within the hydrophobic interior, to the polar aqueous environment. This change in microenvironment results in measurable alterations in tryptophan's spectroscopic properties, exhibiting a red shift in λ_max_ to values ranging from 342 to 345 nm (Figure 4), which is evident after silanization, amidation, and thiourea treatment. However, glutaraldehyde cross‐linking of BSY and HBSY displayed an increasing pattern in *H*_0_ with no red shift in λ_max_ to 345 nm.

#### 3.1.4 FTIR and CD Results

**FIGURE 5:**
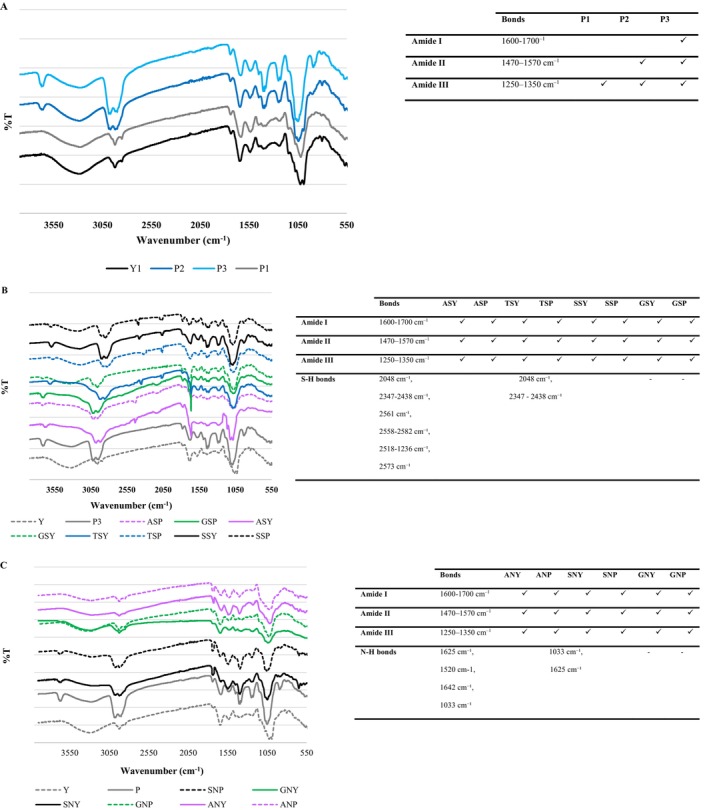
FTIR spectra of BSY and HBSY (A), thiolated BSY and HBSY (B), and aminated BSY and HBSY (C). Y, Yeast, P1, Hydrolyzed yeast with 4.6% degree of hydrolysis, P2, Hydrolyzed yeast with 7.8% degree of hydrolysis, P3, Hydrolyzed yeast with 9.2% degree of hydrolysis. ANP, Amination of hydrolyzed yeast by amidation; ANY, Amination of yeast by amidation; ASY, Thiolation of yeast by amidation; GNP, Amination of hydrolyzed yeast by glutaraldehyde cross‐linking; GNY, Amination of yeast by glutaraldehyde cross‐linking; GSP, Thiolation of hydrolyzed yeast by amidation; GSP, Thiolation of hydrolyzed yeast by glutaraldehyde cross‐linking; GSY, Thiolation of yeast by glutaraldehyde cross‐linking; SNP, Amination of hydrolyzed yeast by silanization; SNY, Amination of yeast by silanization; SSP, Thiolation of hydrolyzed yeast by silanization; SSY, Thiolation of yeast by silanization; TSP, Thiolation of hydrolyzed yeast by thiourea; TSY, Thiolation of yeast by thiourea.

Figure 5 illustrates the FTIR spectra of the BSY, HBSY, aminated, and thiolated‐ BSY and HBSY samples. Peaks observed in the infrared spectrum of yeast were assigned as follows: a broad band at 3407 cm^−1^ to O—H/N—H stretching; 1460 cm^−1^ to C—N stretching; and 1387 cm^−1^ to N—H bending.

Proteins comprise different areas, including (a) the amide I band (1600–1700 cm^−1^), a prominent spectral feature that predominantly arises from C=O stretching, making it highly sensitive to protein secondary structure. (b) The amide II band (1470–1570 cm^−1^) also plays a crucial role, exhibiting a complex vibrational pattern composed of N—H bending and C—N stretching and consequently showing conformational sensitivity. (c) And also amide III (1250–1350 cm^−1^) arises mainly from N—H bending (in‐plane) vibrations, and C—N stretching vibrations of the peptide bond (—CONH—). Moreover, the amide III region (1250–1350 cm^−1^) is particularly useful because its vibrations reflect the coupling between N—H bending and C—N stretching, which changes depending on how peptide bonds are oriented. Additionally, its peak position and intensity depend strongly on the protein's secondary structure (e.g., α‐helix in 1300–1340 cm^−1^, is associated with strongly coupled N—H blend and C—B stretch, β‐sheet in 1230–1245 cm^−1^ is related to hydrogen‐bonded and extended conformation, and random coil in 1250–1270 cm^−1^ is attributed to less ordered structure Guo et al. 2022).

Enzymatic hydrolysis caused more substantial changes in the amide I, II, and III bands, indicating alterations in the secondary structure of BSY as 42.1% in P1, 92.97% in P2, and 118.16% in P3 (Figure 5A). In this regard, a change in the amide I band (1660–1650 cm^−1^) indicates alterations in the protein's secondary structure and disruption of the protein structure during hydrolysis. Moreover, an alteration in the amide III band from 1230 cm^−1^ to 1240 cm^−1^ is observed, corresponding to changes in the characteristic absorption peak of β‐sheets, further supporting the structural disruption. Furthermore, changes in the 3200–3500 cm^−1^ region, representing hydrogen bonding, reflect the impact of hydrolysis on the protein's hydrogen bond network. HBSY showed reduced peak intensity in the amide A region (3500–3000 cm^−1^). Furthermore, the characteristic peak at 2927 cm^−1^, located within the 3000–2800 cm^−1^ region, is attributed to C—H stretching vibrations. It indicates the protein's hydrophobic domain (Liu et al. 2022), which was reduced after hydrolysis, implying the disruption of BSY's hydrophobic regions following pepsin treatment and confirming the reduction in *H*_0_. The extent of secondary structure changes correlated with the specific protease used and its preference for particular cleavage sites. Gao et al. (2024) revealed minimal changes in the hydrogen bonding region (around 3627 cm^−1^) in FTIR analysis. This suggests that O—H and N—H bonds in soy protein isolate remained largely intact after hydrolysis by various proteases. However, significant red shifts were observed in the 1200–1600 cm^−1^ region, attributed to alterations in C—H group reactivity (Gao et al. 2024).

**FIGURE 6:**
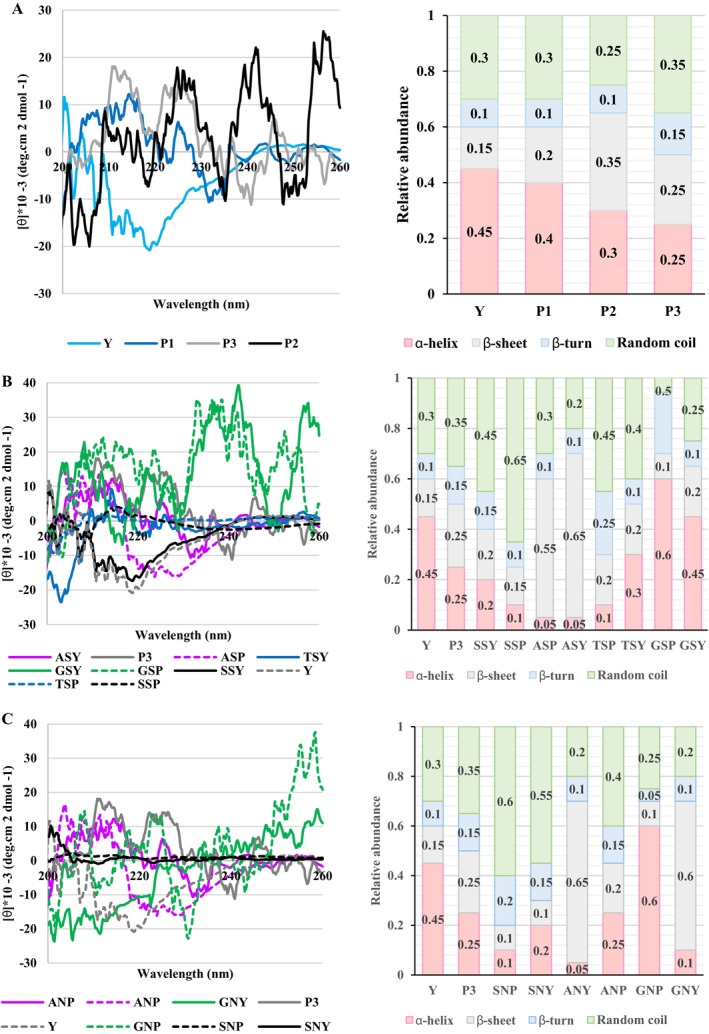
CD spectra of BSY and HBSY (A), thiolated BSY and HBSY (B), and aminated BSY and HBSY (C). Y, Yeast, P1, Hydrolyzed yeast with 4.6% degree of hydrolysis, P2, Hydrolyzed yeast with 7.8% degree of hydrolysis, P3: Hydrolyzed yeast with 9.2% degree of hydrolysis. ANP, Amination of hydrolyzed yeast by amidation; ANY, Amination of yeast by amidation; ASY, Thiolation of yeast by amidation; GNP, Amination of hydrolyzed yeast by glutaraldehyde cross‐linking; GNY, Amination of yeast by glutaraldehyde cross‐linking; GSP, Thiolation of hydrolyzed yeast by amidation; GSP, Thiolation of hydrolyzed yeast by glutaraldehyde cross‐linking; GSY, Thiolation of yeast by glutaraldehyde cross‐linking; SNP, Amination of hydrolyzed yeast by silanization; SNY, Amination of yeast by silanization; SSP, Thiolation of hydrolyzed yeast by silanization; SSY, Thiolation of yeast by silanization; TSP, Thiolation of hydrolyzed yeast by thiourea; TSY, Thiolation of yeast by thiourea.

Pepsin hydrolysis, in particular, resulted in more significant changes, likely due to its targeting of hydrophobic amino acid residues within the α‐helix, forming more flexible structural units as revealed in CD results (Figure 6). The α‐helix reduced 11.1%, 33.3%, and 44.4% after pepsin hydrolysis in P1, P2, and P3, respectively. The β‐sheet increased 33.3%, 133.3% and 66.6% in P1, P2, and P3, respectively (Figure 6). In line with the present study, Wang et al. (2025) noted that significantly reducing the α‐helix and β‐sheet content of the protein led to a substantial increase in the random coil structure. Moreover, this observation aligns with the findings of Bing et al. (2024) and Tan et al. (2022), who reported a similar disruption of α‐helix and β‐turn structures in the protein after protease treatment. Similarly, Zang et al. (2019) noted changes in rice bran protein, and Feng et al. (2024) stated an alteration in walnut protein, corroborating our findings. However, Xingfeng Xu et al. (2016) reported that enzymatic hydrolysis led to a decrease in β‐sheet content. A minor decrease was observed in β‐turn content. Conversely, the content of random coil and α‐helix structures increased. This is consistent with the relative stability of β‐sheets compared to the greater flexibility of α‐helices, β‐turns, and random coils.

According to Figure 5B, the S—H stretching vibration of the thiol group is observed in the infrared spectra as the appearance of bands at 2048 cm^−1^ and 2347–2438 cm^−1^. The intensity band in thiolated HBSY was greater than that in thiolated BSY. In addition, the intensity band in silanization was significantly higher than that of other thiolation methods. Moreover, none of these bands was observed with the glutaraldehyde cross‐linking method. The study conducted by Qiu et al. (2020) observed that the S—H peak at 2561 cm^−1^ arises from thiol group aggregation and hydrogen bonding. Kuodis et al. (2020) identified a band near 2558 cm^−1^, which shifts to 2582 cm^−1^ upon dissolution of the thiol compound in water, due to strong hydrogen bonding interactions. In a study conducted by Ebrahimnia et al. (2025), it was reported that weak peaks at approximately 2518 cm^−1^ and 1236 cm^−1^ in thiolated gelatin, absent or significantly weaker in unmodified gelatin, are attributable to S—H and C—S bond vibrations, respectively, confirming the successful thiol modification. Moreover, the enhanced intensity of the band at 2573 cm^−1^ in thiolated gelatin supports the successful incorporation of thiol groups. The CD spectra of thiolated samples revealed that after thiolation (except GSY and GSP), the content of α‐helix and β‐sheet significantly decreased, and the level of β‐turn and random coil increased. Compared to TSP and ASP, SSP led to the most significant decrease in both α‐helix (60%) and β‐sheet (40%) content. TSP showed a similar reduction in α‐helix (60%) but a minor β‐sheet content (20%), while ASP exhibited the highest reduction in α‐helix (80%) but a significant increase in β‐sheet (120%). As can be shown in the CD results, the content of α‐helix and β‐sheet decreased, resulting in a conversion to a random coil structure (Figure 6). The increase in random coil was as follows: SSP (116.66%) > SSY/TSP (28.57%) > TSY (14.28%). The difference between TSP and SSY is the higher level of α‐helix in SSY rather than TSP (Figure 6). Although ASP and ASY depicted the highest reduction in the α‐helix (60%), the random coil also reduced.

FTIR analyses investigated the chemical states of amine groups and their influence on surface compatibility. Following amination, the spectrum shows an enhanced N—H feature with bands at 1033 cm^−1^ and 1625 cm^−1^ (Figure 5C). A band at 1582 cm^−1^ appearing as a shoulder on the adsorbed water deformation mode was assigned to NH_2_ bending vibrations. A broad band between 1540 and 1490 cm^−1^ corresponded to asymmetric NH_3_^+^ deformation vibrations. Amine functional groups exhibit characteristic absorption bands in FTIR spectra, typically appearing in the 3500–3300 cm^−1^, 1650–1590 cm^−1^, and 1100–1000 cm^−1^ regions, as detailed in standard spectral interpretation manuals. Following amination, the position of the amide A, I, and II band peaks exhibited a slight shift, suggesting the conformational changes from α‐helix, β‐sheet, and β‐turn content to the random coil structure, which was in line with Ma et al. (2025) who modified collagen fibrils with monoethanolamine or diethanolamine. The absorption peaks at 1625 cm^−1^ and 1520 cm^−1^ are used to quantify the amine functional group (Kamruzzaman et al. 2018). Amination modification of the lignin was successfully achieved, as indicated by the generation of a new band at 1642 cm^−1^ (C≡N stretch) and a peak at 1033 cm^−1^ (C—N stretch) (Jia et al. 2024).

Following amination, ANY resulted in the highest reduction (80%), followed by SNP (60%) and SNY (20%). Similarly, β‐sheet was reduced in SNP and SNY (60%), followed by ANP (20%). The CD data show a decrease in both α‐helix and β‐sheet, corresponding to a shift toward a random coil conformation. The random coil increased by approximately 71.42% (SNP) > 57.14% (SNY) > 14.28% (ANP) (Figure 6).

#### 3.1.5 SEM Analysis

**FIGURE 7:**
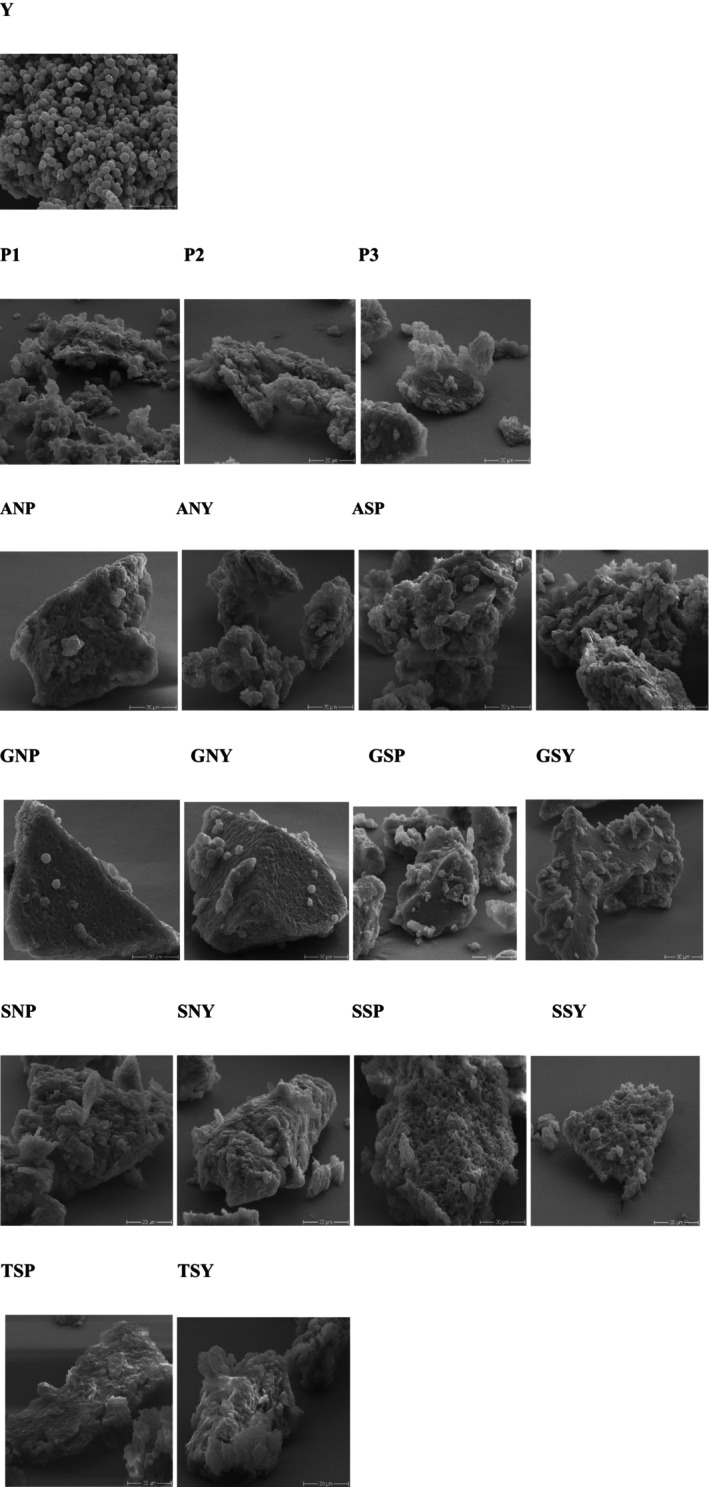
SEM analysis of Y, Yeast, P1, Hydrolyzed yeast with 4.6% degree of hydrolysis, P2, Hydrolyzed yeast with 7.8% degree of hydrolysis, P3, Hydrolyzed yeast with 9.2% degree of hydrolysis. ANP, Amination of hydrolyzed yeast by amidation; ANY, Amination of yeast by amidation; ASY, Thiolation of yeast by amidation; GNP, Amination of hydrolyzed yeast by glutaraldehyde cross‐linking; GNY, Amination of yeast by glutaraldehyde cross‐linking; GSP, Thiolation of hydrolyzed yeast by amidation; GSP, Thiolation of hydrolyzed yeast by glutaraldehyde cross‐linking; GSY, Thiolation of yeast by glutaraldehyde cross‐linking; SNP, Amination of hydrolyzed yeast by silanization; SNY, Amination of yeast by silanization; SSP, Thiolation of hydrolyzed yeast by silanization; SSY, Thiolation of yeast by silanization; TSP, Thiolation of hydrolyzed yeast by thiourea; TSY, Thiolation of yeast by thiourea. Scale bars are 20 μm.

Unmodified yeast cells exhibited an elliptical shape with a smooth surface. As shown in Figure 7, following enzymatic hydrolysis, the initially insoluble BSY was observed to be fragmented into small, spherical particles exhibiting a lamellar structure with a wrinkled surface. This morphological change indicates disassembly and unfolding of insoluble BSY aggregates. The resulting more flexible protein structure likely enhances solubility in aqueous systems by increasing the number of available sites for protein‐water interactions. This was in agreement with Gao et al. (2024).

A fragmented morphology is observed after thiolation and amination, suggesting enzymatic cleavage of peptide bonds and disruption of the protein's tertiary and secondary structure, as described in Section 3.1.4. Thiolated and aminated samples display a loose, heterogeneous, flaky structure. These structural changes are expected to impact the resulting physicochemical and functional properties significantly (Wang, Wang, Wang, et al. 2021). Silanization and amidation resulted in noticeable cell disruption and a roughened cell surface, creating a sheet‐like state on the surface, which is attributed to the application of highly concentrated acids and bases. The cells thiolated by silanization and thiourea experienced significant shrinkage, with some even rupturing. Glutaraldehyde cross‐linking resulted in noticeable cell adhesion (Figure 7), likely leading to some adsorption sites being encapsulated within the clusters and the emergence of granular protrusions on the surface of the glutaraldehyde cross‐linked cells, as well as very compact and fused structures.

### 3.2 Effect of Limited Enzymatic Hydrolysis on Functional Properties

BSY and HBSY surfaces underwent substantial changes following the introduction of amino groups (NH_2_^+^) and thiol groups (S^−^) due to an increase in the density of positively and negatively charged groups. This strategy offers a promising approach to improving protein functionalities (Pinheiro et al. 2023). Silanization‐produced thiolation and amination samples were selected to evaluate their functional and antioxidant properties. The HBSY with DH 9.2% (P3) also represented the highest degree of thiolation and amination.

#### 3.2.1 Solubility

**FIGURE 8:**
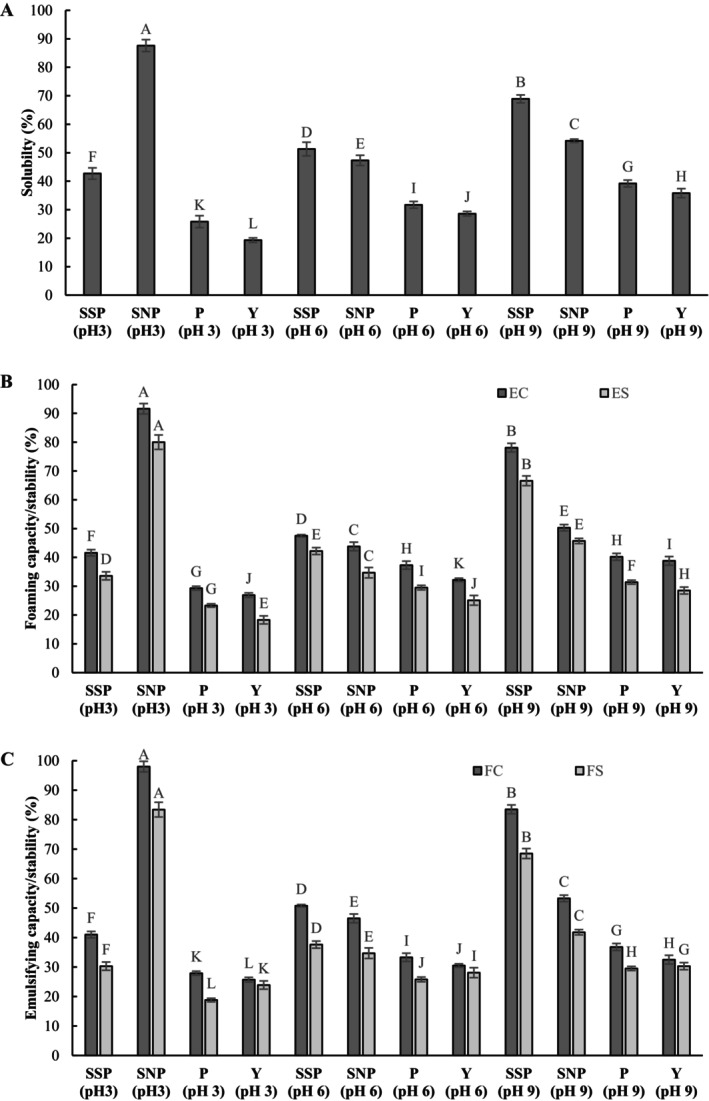
Solubility (A), foaming capacity (FC) and stability (FS) (B), emulsifying capacity (EC) and stability (ES) (C) of SSP3 (Thiolated HBSY by silanization at pH 3); SNP3 (Aminated HBSY by silanization at pH 3); P3 (Hydrolyzed yeast at pH 3); Y3 (Brewers spent yeast at pH 3); SSP6 (Thiolated HBSY by silanization at pH 6); SNP6 (Aminated HBSY by silanization at pH 6); P6 (Hydrolyzed yeast at pH 6); Y6 (Brewers spent yeast at pH 6); SSP9 (Thiolated HBSY by silanization at pH 9); SNP9 (Aminated HBSY by silanization at pH 9); P9 (Hydrolyzed yeast at pH 9); and Y9 (Brewers spent yeast at pH 9). Values are the means ± SD of three replicate experiments. The capital letters in each column indicate significant differences (*p* < 0.05).

**FIGURE 9:**
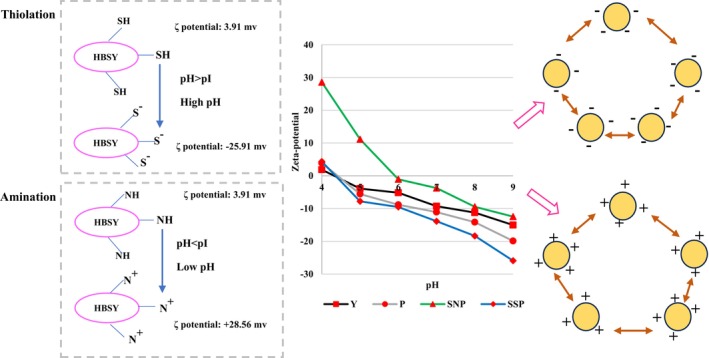
The effect of thiol and amino groups on ζ potential and functional properties.

Solubility is a critical determinant of protein functionality, including emulsification and foaming (Guo et al. 2022). Enzymatic hydrolysis significantly enhances the solubility of HBSY, thiolated‐ and aminated HBSY across the investigated pH ranges (Figure 8A). HBSY (28.6%–39.2%) exhibited significantly higher solubility than BSY (19.3%–31.7%). This enhanced solubility is likely a consequence of several factors: (1) the reduced molecular weight of the protein fragments and the structural unfolding that exposes previously hidden polar groups and charged residues (SEM Section 2.8). This increased exposure led to electrostatic repulsion (Figure 9) and random coil formation (CD results) (Figure 5), preventing the formation of large, insoluble aggregates and facilitating interactions with water molecules through hydrogen bonding and electrostatic forces, which enhances solubility (Zheng et al. 2015). (2) The reduced *H*_0_ facilitates the solubility of HBSY with increasing %DH. The balance of hydrophobic and hydrophilic regions on a protein's surface dictates its interactions with water and other proteins, thereby influencing solubility. Surface charge is a significant determinant of protein solubility, although other factors, such as molecular size, charge distribution, and hydrophobicity, also play a role. Minimal solubility was observed for BSY and HBSY at pH 3, which is close to their respective isoelectric points of 4.4 and 4.3 (Table 2). This low solubility results from reduced electrostatic repulsion between protein molecules at their net charge of zero. The improved solubility at higher pH is likely due to increased electrostatic repulsion and more favorable interactions between the protein and solvent molecules. In a study by Vogelsang‐O'Dwyer et al. (2023) and Segura‐Campos et al. (2012), the lowest solubility was observed in pI with the lowest zeta‐potential.

**TABLE 2:** Isoelectric pH of spent yeast after thiolation and amination.

Sample	pI
Y	4.4
P3	4.3
ASP	3.5
ASY	3.8
TSP	3.3
TSY	3.7
SSP	3.1
SSY	3.3
GSP	4.4
GSY	4.4
ANP	5.2
ANY	4.9
SNP	5.3
SNY	5.1
GNP	4.4
GNY	4.4

Thiolation and amination increased protein solubility, likely due to the feasibility of H_2_ bonding of water with —SH and —NH groups in these compounds. Moreover, thiolated and aminated BSY/HBSY presented lower *H*_0_. Furthermore, the formation of more random coil structures is another reason for enhancing the solubility in thiolated and aminated BSY/HBSY. Solubility was also pH‐dependent, with increased solubility at acidic pH for aminated HBSY and at basic pH for thiolated HBSY (Gao et al. 2024). In line with our study, the pI for spent brewer's yeast protein is reported to be 4.4 (Velez‐Erazo et al. 2021), which was reduced to 4.3 (HBSY), 3.8 (ASY), 3.5 (ASP), 3.7 (TSY), 3.3 (TSP), 3.2 (SSY), and 3 (SSP). Thiolation of proteins can alter their net charge in two ways: by adding a charged low‐molecular‐weight thiol or by modifying a negatively charged sulfhydryl group within the protein itself. An enhancement in the solubility index of gelatin following thiolation has been reported by Ebrahimnia et al. (2025), whose pI was reduced by approximately 4.5, consistent with our study findings. This observation is attributed to the disruption of intermolecular hydrogen bonding and the consequent reduction in crystallinity resulting from incorporating hydrophilic thiol groups. The hydrophilicity of the thiol groups in the nanofibers caused significant swelling due to hydrogen bonding with water (Figures 8A and 9).

After amination, the pI increased from 4.4 (BSY) to 4.9 (ANY), 5.2 (ANP), 5.0 (SNY), and 5.3 (SNP). Ma et al. (2025) reported that the increased pI of monoethanolamine‐ and diethanolamine‐modified collagen fibers (0.48 and 0.61 units, respectively) results from the efficient addition of amino groups to the collagen side chains.

The optimal solubility characteristics were observed for the thiolated and aminated samples at pH 9 (68.9 ± 1.4) and pH 3 (87.6 ± 2.1), respectively, reflecting the highest levels of free *N* ^+^ and S^−^ among all samples analyzed.

#### 3.2.2 Emulsifying Activity (EA) and Emulsion Stability (ES)

EA and ES are critical interfacial protein traits. EA reflects a protein's ability to create and maintain an emulsion, whereas ES measures the stability of the emulsion dispersion, preventing clumping or aggregation. These amphiphilic molecules stabilize oil‐in‐water emulsions by forming interfacial films around oil droplets, which are governed by emulsifying capacity and stability (Feng et al. 2024).

Figure 8B summarizes the EA and ES produced from BSY, HBSY, aminated‐ and thiolated HBSY at pH levels 3, 6, and 9. As illustrated in Figure 8B, the thiolated and aminated HBSY exhibited significantly higher EA and ES values (*p* < 0.05) compared to BSY and HBSY, indicating an improvement in emulsifying properties resulting from surface modification through thiolation and amination. The EA increased from 26.9–38.8 (BSY) to 29.3–40.2 (HBSY). Interestingly, the EA increased progressively from 41.6 ± 0.8 (pH 3) to 78.1 ± 1.6 (pH 9) for thiolated and from 43.8 ± 1.3 (pH 6) to 91.6 ± 0.9 (pH 9) for aminated HBSY, respectively. A similar pattern was observed for ES in the range of 33.6 ± 0.4 to 66.6 ± 1.8 (for thiolated HBSY) and 34.7 ± 0.9 to 80.0 ± 0.8 (for aminated HBSY).

Enzyme treatment enhanced the EA of HBSY, likely due to the smaller peptide molecules in the resulting HBSY. This hydrolysis process broke BSY into smaller peptides with lower molecular weights, which could swiftly migrate to the oil–water interface (SEM Section 2.8, Figure 7). These smaller peptides diffused more uniformly during homogenization, occupying a larger interfacial area and facilitating rearrangement and adsorption. Moreover, surface hydrophobicity is a key determinant of protein techno‐functionality, influencing emulsifying and foaming capabilities. The enhanced EA is related to altered surface hydrophobicity in hydrolyzed proteins, characterized by an increase in hydrophilic carboxyl and amino groups, as illustrated in the hydrophobicity section (Wang, Cheng, et al. 2022). Furthermore, this flexibility enables adsorption and rearrangement at the oil–water interface, leading to a stable, continuous film around oil droplets and improved ES (Jiang et al. 2015). As discussed in CD spectra, the increase in random coil was observed as follows: SNP > SSP > P > Y. The greater random coil content in SNP and SSP compared with HBSY and BSY indicates a transition to a more disordered and flexible conformation. Similarly, ES measurements confirmed that HBSY has an enhanced ability to stabilize oil–water interfaces over time compared to BSY. This improvement is attributed to the enhanced flexibility of HBSY's secondary structure, which promotes adsorption at the oil–water interface. Hydrolyzed HBSY is also a promising emulsifier for improving emulsion stability (Gao et al. 2024). Thiolated and aminated HBSY show considerable potential as hydrophilic emulsifiers. Previous studies have demonstrated a correlation between increased solubility and improved emulsifying properties of jackfruit leaf protein hydrolysates (Calderón‐Chiu et al. 2021) and walnut protein hydrolysates (Jin et al. 2020). Studies conducted by Feng et al. (2024) and Ghribi et al. (2015) indicated that prolonged enzymatic digestion increased EA but decreased ES, probably due to reduced interfacial viscoelasticity from smaller peptides' decreased interaction at the oil–water interface. This differs from Jing Wang, Liu, et al. (2021), who found that pepsin hydrolysis increased both EA and ES of gluten‐1.

SSP (pH 9) and SNP (pH 3) exhibited zeta‐potentials of −25.91 mV and +28.56 mV, respectively, as measured using a nanoPartica SZ‐100 instrument (Horiba Ltd., Japan) (Figure 8). It is established that SSP and SNP indicate stronger electrostatic repulsion than BSY and HBSY, leading to enhanced droplet stability at basic and acidic pH (Amiri et al. 2024). The zeta‐potential, representing the net surface charge of oil droplets in emulsions, was measured from their electrophoretic mobility and plotted against pH. A charged layer formed by the protein emulsifier on the droplet surface provides a pH‐dependent electrostatic repulsion against aggregation with other droplets. Therefore, the net surface charge of oil droplets serves as a key parameter for predicting ES. Elevated surface potentials create a high‐energy barrier, resulting in repulsion between adjacent droplets and a stable emulsion. Theoretically, emulsions with a zeta‐potential greater than | ±30 mV| are considered stable. Emulsions using BSY and HBSY showed good stability at pH < 4.3–4.4 and pH > 4.3–4.4. At pH 4.3, near HBSY's and 4.4 BSY's theoretical pI, the net protein charge was nearly zero, making these emulsions unstable. The absence of sufficient electrostatic repulsion at the pI promotes flocculation and potential coalescence, thereby accelerating creaming unless a continuous network of flocculated droplets is established (Ma et al. 2011). After thiolation and amination, the pI shifted to 3.1–3.8 and 4.9–5.3, respectively, as shown by the zeta‐potential. Stabilized emulsions moved to the alkaline (pH 9) and acidic side (pH 3), which represent the highest free S^−^ and *N* ^+^content, indicating that the SSP and SNP at the emulsion interface carried enough net charge to provide electrostatic repulsion and stabilize the emulsions through alkaline and acidic pHs, respectively.

Chang et al. (2017) demonstrated that thiol modification enhanced the foaming capacity and emulsifying stability, particularly at pH levels of 8 and 9, in fibrillated whey protein isolate. Thiolation increased zeta‐potential values, indicating enhanced electrostatic repulsion.

#### 3.2.3 Foaming Capacity (FC) and Foam Stability (FS)

Pepsin‐mediated enzymatic hydrolysis significantly enhanced the FC of BSY protein from 25.7%–32.5% to 27.9%–36.8% for HBSY (*p* < 0.05; Figure 8C). This improvement is attributed to the enhanced solubility and flexibility of HBSY, which contribute to improved diffusion and adsorption at the air‐water interface. This consequently leads to the formation of films characterized by superior interfacial viscoelasticity and enhanced bubble formation capabilities (Liu et al. 2022). HBSY demonstrated enhanced FC compared to BSY across both acidic and alkaline pH ranges. This observation is likely attributable to low peptide solubility and increased electrostatic repulsion due to pH values that are far from the pI. Hydrolysis yielded significantly lower FS, reaching a maximum of 18.8%–29.5% for HBSY compared to 23.9%–30.5% for BSY. A negative correlation was observed between FS and DH%, suggesting that excessive hydrolysis compromises the structural integrity required for stable foam formation. This is consistent with the established understanding that larger peptides facilitate the formation of more substantial, more elastic interfacial films around air bubbles, whereas smaller peptides are less effective in this regard (Phongthai et al. 2016; Wouters et al. 2016). This observation is further supported by Feng et al. (2024), who demonstrated a positive correlation between high‐molecular‐weight peptides and foam stability in walnut protein through limited enzymatic hydrolysis. An investigation into the influence of pH on the foaming properties of BSY, HBSY, and their thiolated and aminated derivatives revealed a pH‐dependent effect (Figure 9). The FC (41%–83.5%) and FS (30.3%–68.7%) of thiolated HBSY were higher than those of BSY and HBSY. Similarly, an increasing trend was observed for FC (from 98.1% to 46.5%) and FS (from 83.4% to 34.7%) of aminated HBSY. The thiolated HBSY demonstrated higher FC and FS at alkaline pH (83.5% and 68.7%), while the aminated HBSY depicted higher FC and FS at acidic pH (98.3% and 83.4%), respectively.

The enhanced FC and FS of thiolated and aminated BSY/HBSY results from the introduction of polar —SH and —NH groups. These groups increase the protein's capacity for hydrogen bonding with water, reducing hydrophobicity (as indicated by lower *H*_0_ values) and promoting a more disordered random coil conformation, all of which contribute to improved solubility. The superior foamability and stability of SNP compared to SSP can be attributed to differences in surface activity. SNP's lower surface activity results in lower surface tension, making foam formation easier (Majzoobi et al. 2012).

### 3.3 Effect of Limited Enzymatic Hydrolysis on Antioxidant Activity

#### 3.3.1 DPPH Radical Scavenging Activity

The DPPH radical scavenging activity of BSY, HBSY, aminated‐, and thiolated HBSY (at %DH 9.2%) was evaluated as a function of protein concentration. A positive correlation was observed between concentration and DPPH radical scavenging activity for all samples (Table 3). Specifically, increasing the concentration from 1.0 to 3.0 mg/mL resulted in percentage increases ranging from 5.6% to 11.7% in BSY and from 12.9% to 24.6% in HBSY. HBSY samples exhibited the highest DPPH radical scavenging activity at a concentration of 3.0 mg/mL. Thiolated and aminated HBSY significantly (*p* < 0.05) enhanced DPPH radical scavenging to 33.6%–79.1% and 21.9%–52.7%, respectively.

**TABLE 3:** The antioxidant properties of BSY, HBSY, and thiolated and aminated HBSY by silanization.

Concentration (mg/mL)	Samples	DPPH radical scavenging activity (%)	Reducing power	OH° radical scavenging activity (%)
1	SSP	33.6 ± 0.4^E^	0.37 ± 0.03^E^	32.4 ± 0.5^D^
	SNP	21.9 ± 0.2^F^	0.28 ± 0.04^F^	23.7 ± 0.2^E^
	P	12.9 ± 0.3^I^	0.19 ± 0.02^H^	17.4 ± 0.4^H^
	Y	5.6 ± 0.2^L^	0.08 ± 0.01^K^	8.7 ± 0.3^K^
2	SSP	53.3 ± 0.6^C^	0.58 ± 0.03^C^	48.5 ± 0.6^B^
	SNP	38.3 ± 0.5^D^	0.41 ± 0.05^D^	37.1 ± 0.4^C^
	P	17.8 ± 0.5^H^	0.26 ± 0.01^G^	19.3 ± 0.3^G^
	Y	8.3 ± 0.2^K^	0.11 ± 0.01^J^	9.2 ± 0.1^J^
3	SSP	79.1 ± 0.7^A^	0.92 ± 0.05^A^	60.8 ± 0.8^A^
	SNP	52.7 ± 0.6^B^	0.69 ± 0.04^B^	46.9 ± 0.4^B^
	P	24.6 ± 0.5^G^	0.31 ± 0.02^F^	22.3 ± 0.5^F^
	Y	11.7 ± 0.1^J^	0.13 ± 0.01^I^	10.1 ± 0.2^I^

*Note:* The values are the means ± SD of three replicate experiments. The capital letters in each column indicate significant differences (*p* < 0.05).

Abbreviations: P, hydrolyzed yeast; SNP, amination by silanization of HBSY; SSP, thiolation by silanization of HBSY; Y, yeast.

HBSY exhibited enhanced antioxidant properties compared to BSY. The observed effects resulted from the generation of antioxidant peptides and the discharge of hydrophobic amino acids through enzymatic digestion. Gao et al. (2024) indicated that variations in antioxidant activity among soy protein hydrolysates produced using different enzymes reflect the distinct substrate specificities of these enzymes. Different enzymes displayed different cleavage patterns. Pepsin hydrolyzes peptide bonds within protein domains. Bromelain's activity focuses on amino acids containing reactive groups, such as inertial and imidazolyl, which are known for their antioxidant properties. Trypsin, on the other hand, preferentially cleaves aromatic amino acids characterized by their high oxidative potential (Gao et al. 2024). Calderón‐Chiu et al. (2021) identified a dose‐dependent correlation between the amount of jackfruit leaf protein hydrolysates and their antioxidant activity, aligning with the present study's findings. Additionally, whey protein's DPPH radical scavenging activity was enhanced as the DH was augmented. These outcomes suggest that enzymatic hydrolysis disrupts the tightly packed spatial arrangement of the BSY protein, thereby facilitating the exposure of active peptides that can interact with oxidants (Singh et al. 2021). Similarly, Phongthai et al. (2016) demonstrated that a higher DH in rice bran protein corresponded to enhanced DPPH radical scavenging effectiveness.

Thiolate (—SH) and amino (NH) groups can react with electrophilic reactive species through two primary mechanisms: nucleophilic substitution or Michael addition to an α,β‐unsaturated carbonyl compound, resulting in the formation of a covalent adduct. Thiolated and aminated HBSY display significantly more vigorous, concentration‐dependent radical scavenging activity.

The reactivity of a thiol group depends on its protonation state. The protonated form (—SH) is not particularly reactive. However, the deprotonated form, the thiolate anion (S^−^), exhibits high electron density, rendering it a strong nucleophile. Several factors govern the inherent reactivity of a thiol group, most notably its accessibility and pKa. The pKa is the pH at which 50% of the thiol is deprotonated (Ebrahimnia et al. 2025). Thiols with lower pKa values exist primarily as deprotonated thiolate anions, increasing their susceptibility to modification by reactive species such as electrophiles (Ebrahimnia et al. 2025). Ebrahimnia et al. (2025) stated that this enhanced activity in the thiolated form is attributed to the formation of disulfide bonds.

DPPH radical scavenging activity was assessed by measuring EC_50_ values. The SSP and SNP fractions showed EC_50_ values of 1.765 and 2.850 mg/mL, respectively. In contrast, the BSY and HBSY exhibited EC_50_ values greater than 3 mg/mL (Table 4).

**TABLE 4:** Antioxidant effect (EC_50_) on DPPH radicals and hydroxyl radicals of BSY, HBSY, and thiolated and aminated HBSY by silanization.

Samples	EC_50_ (mg/mL)	
	DPPH radical scavenging activity (%)	OH° radical scavenging activity (%)
SSP	1.765 ± 0.15	2.194 ± 0.13
SNP	2.85 ± 0.12	> 3
P	> 3	> 3
Y	> 3	> 3

*Note:* The values are the means ± SD of three replicate experiments. The capital letters in each column indicate significant differences (*p* < 0.05).

Abbreviations: P, hydrolyzed yeast; SNP, amination by silanization of HBSY; SSP, thiolation by silanization of HBSY; Y, yeast.

These results are consistent with those of previous studies evaluating the antioxidant properties of compounds containing thiol and amino groups. The study by Takashima et al. (2012) assessed the reactivity of three thiols—cysteine, homocysteine, and glutathione—toward galvinoxyl, DPPH, and peroxyl radicals, finding substantial scavenging activity with a decreasing trend: galvinoxyl > DPPH > peroxyl. Cysteine, homocysteine, and glutathione also mitigated methyl linoleate oxidation in micellar systems. In another study conducted by Güngör et al. (2011), the antioxidant performance of SH antioxidants (N‐acetyl cysteine, 1,4‐dithioerythritol, cysteamine, reduced glutathione, reduced glutathione ethyl ester, cysteine, reduced lipoic acid, and homocysteine) and S‐type antioxidants (oxidized glutathione, lipoic acid, cystine, homocystine, and methionine) reflects a complex interplay of structural characteristics. Their investigation revealed that substituting OH groups with SH groups resulted in a substantial increase in antioxidant properties. Critical determinants include: (i) the number of free —SH groups available for radical scavenging, (ii) the overall conjugation that can influence electron delocalization, (iii) the accessibility of —SH groups to interact with the chromogenic reagent used for detection, and (iv) the electronic and steric effects of substituents proximal to the —SH groups (Güngör et al. 2011). Cerit and Demirkol (2021) observed enhanced antioxidant activity in French fries with cysteine and N‐acetylcysteine.

To investigate the antioxidant properties of amino derivatives, a study on the structure–activity relationships of six novel 4‐methylcoumarins with distinct functional groups (amino, hydroxy, N‐acetyl, acetoxy, and nitro) was conducted. The data revealed that amino substitution provides superior antioxidant protection relative to hydroxyl substitution, markedly reducing lipid peroxidation. The highest activities in both antioxidant and radical scavenging assays were detected in the ortho‐dihydroxy and ortho‐hydroxy–amino coumarins (Tyagi et al. 2005). The findings of Yuan et al. (2022) indicate that amino‐functionalized chitosan possesses superior antioxidant capacity, achieving approximately 90% at the lowest tested concentration (0.10 mg/mL). Considering the high electron‐donating potential of the amino group, substituting it at different positions on honokiol is expected to consistently enhance honokiol's antioxidant capacity as compared to the unsubstituted molecule (Liu et al. 2021). Amino group modification markedly elevates genistein's antioxidant potential, illustrating the greater effectiveness of amino‐substituted analogs (Wang et al. 2019).

#### 3.3.2 Reducing Power

Table 3 illustrates that within the 1.0 to 3.0 mg/mL concentration range, BSY, HBSY, aminated, and thiolated HBSY increased their reducing power, demonstrating a clear dose‐dependent relationship. Specifically, the reducing power of thiolated and aminated HBSY increased by 362%–607% and 300%–580%, respectively, as the concentration rose from 1.0 to 3.0 mg/mL. Singh et al. (2021) and Feng et al. (2024) revealed a significant enhancement in the reducing power of rice bran and walnut proteins following hydrolysis, which aligns with the findings of this research. The nucleophilic nature of thiolate (—S^−^) and amino (—NH_2_ or —NH_3_^+^) groups allows them to react with electrophilic species. However, their reactivity differs significantly. Thiolates are strong nucleophiles that readily attack electrophilic centers, resulting in the formation of thioethers or oxidation. In contrast, amino groups, especially in their unprotonated state, typically form Schiff bases with aldehydes; however, they can also participate in a wider range of reactions (Segura‐Campos et al. 2012).

#### 3.3.3 Hydroxyl Radical Scavenging Activity

Regarding hydroxyl radical scavenging activity (Table 3), aminated and thiolated HBSY demonstrated a more significant scavenging potential than HBSY and BSY. The scavenging activity for hydroxyl radicals for thiolated and aminated HBSY within the concentration range of 1.0–3.0 mg/mL was observed to be 32.4%–60.8% and 23.7%–46.9%, respectively. All of these values exceeded those of the BSY, which ranged from 8.7% to 10.1%, and HBSY, which ranged from 17.4% to 22.3%. The SSP fractions exhibited the lowest hydroxyl radical scavenging EC_50_ values (2.194 mg/mL) compared to other samples (Table 4). These results align with the study's outcomes by Shahi et al. (2020) and Wang, Wang, Chen, et al. (2021), which also reported enhanced hydroxyl radical scavenging activity in proteins hydrolyzed by pancreatin, alcalase, and pepsin. Furthermore, existing literature has indicated that hydroxyl radicals play a crucial role in the lipid peroxidation process (Feng et al. 2024; Liu et al. 2022; Wang, Wang, Chen, et al. 2021; Wang, Liu, et al. 2021). The enhanced hydroxyl radical scavenging activity of aminated and thiolated HBSYs suggests their potential application in preventing lipid peroxidation in food products.

## 4 Conclusion

This study demonstrates the successful synthesis of thiol‐ and amino‐functionalized hydrolyzed BSY via different reagents. Hydrolysis decreased the hydrophobicity of BSY, enabling efficient modification by introducing thiol and amino groups through silanization rather than other modifying agents. The relationships between %DH and surface groups contrasted sharply with those of *H*_0_. %DH showed strong positive correlations with thiol (*r* = 0.99, *p* < 0.05) and amino (*r* = 0.99, *p* < 0.05) groups, while *H*_0_ exhibited strong negative correlations with these same groups (thiol: *r* = −0.94, *p* < 0.05; amino: *r* = −0.97, *p* < 0.05). This process altered the HBSY structure, reducing the α‐helix content and increasing the random coil content, thereby impacting its functionality. The thiolated and aminated HBSY significantly improved solubility, foaming, and emulsifying properties compared to unmodified HBSY across all pH ranges, particularly at alkaline pH for thiolated HBSY and acidic pH for aminated HBSY. Zeta‐potential increased in alkaline and acidic pHs following thiolation and amination. The antioxidant properties increased as follows: thiolated HBSY > aminated HBSY > HBSY > yeast. This research highlights the significant potential of spent brewer's yeast functionalization as a sustainable and economically viable approach to waste reduction in the brewing industry. Through thiol and amino modifications, SBY is transformed into high‐value materials applicable in diverse sectors, including food science, pharmaceuticals, and industrial processes. The resulting products exhibit enhanced properties, such as improved stability and shelf life, and demonstrate functional capabilities as emulsifiers, beverage foaming agents, mycotoxin and microbial adsorbents, drug delivery agents, and fermentation substrates. This approach not only minimizes environmental impact but also creates new economic opportunities by transforming a waste by‐product into valuable resources.

## Author Contributions

**Elahe Abedi:** writing – original draft, conceived and designed the research, and analyzed the data conducted experiments. **Philip C. Wietstock:** writing – original draft, conceived and designed the research, and analyzed the data. **Brian Gibson:** writing final draft.

## Ethics Statement

The authors have nothing to report.

## Conflicts of Interest

The authors declare no conflicts of interest.

## Data Availability

The data that support the findings of this study are available from the corresponding author upon reasonable request.
